# Recovery patterns in patients undergoing revision surgery of the primary knee prosthesis

**DOI:** 10.1186/s40634-021-00436-w

**Published:** 2021-12-16

**Authors:** W. Y. Liu, M. C. van der Steen, R. J. A. van Wensen, R. W. T. M. van Kempen

**Affiliations:** 1 Department of Orthopaedic Surgery & Trauma, Catharina Hospital, P.O. box 1350, 5602 ZA Eindhoven, the Netherlands; 2 Department of Orthopaedic Surgery & Trauma, Máxima MC, Eindhoven, the Netherlands

**Keywords:** Knee arthroplasty, Revision arthroplasty, Clinical outcomes, Patient reported outcome measures

## Abstract

**Purpose:**

Despite good survival rates of revised knee prostheses, little is known about recovery trajectories within the first 12 months after surgery. This retrospective observational study explored recovery trajectories in terms of pain, function and quality of life in patients after revision knee arthroplasty over 12 months.

**Methods:**

Eighty-eight revision knee arthroplasty patients rated changes in daily physical functioning using the anchor question (0: very much worsened; 7: very much improved). Patient reported outcome measures (PROMs) of pain (range 0–10), function (Oxford Knee Score) and quality of life (EQ-5D-3L) were assessed preoperatively, at 3 and 12 months postoperatively. Four recovery trajectories were identified using the anchor question at 3 and 12 months postoperatively: no improvement, late improvement, early improvement, and prolonged improvement. Repeated measures ANOVA was conducted with recovery trajectories as dependent variable and PROM assessments as independent variables.

**Results:**

Sixty percent reported improvement in daily physical functioning at 12 months postoperatively. Age and reason for revision differed between groups. Pain, function and EQ-5D-3L differed between groups over time. Late and prolonged improvement groups improved on all PROMs at 12 months. The early improvement group did not report improvement in daily physical functioning at 12 months, while improvements in function and pain during activity were observed.

**Conclusions:**

Different recovery trajectories seem to exist and mostly match PROMs scores over time. Not all patients may experience beneficial outcome of revision knee arthroplasty. These findings are of importance to provide appropriate information on possible recovery trajectories after revision knee arthroplasty to patients.

**Level of evidence:**

III

**Supplementary Information:**

The online version contains supplementary material available at 10.1186/s40634-021-00436-w.

## Introduction

Total knee arthroplasty (TKA) is a highly successful procedure to alleviate pain, and improve function and health related quality of life in patient with knee osteoarthritis [5, 32]. The number of TKA procedures is growing worldwide, with an expected future increase of 143% by 2050 in the United States. This is mainly due to the aging population, increasing rates of obesity, and more TKAs performed in younger patients [14, 20, 21]. Register data report overall 10-year revision rates of TKA of 6.2% (range: 4.9–7.8%) [22]. Consequently, the number of revision procedures is expected to rise as well, with increasing cost burden on the healthcare system [16, 25, 29].

Revision TKA is a challenging, complex procedure [31] with more complications and reoperations needed as compared to primary procedures [30]. Despite this demanding procedure, improvements in pain, function, and quality of life have been reported using patient reported outcome measures (PROMs) [11, 12]. These PROMs are valuable tools to evaluate patient-centered outcomes after treatment, and may be used to identify recovery trajectories of patients after knee arthroplasty within the first 12 months after treatment [7, 8, 19, 24].

Recovery trajectories based on pain and function in primary knee arthroplasty patients have been associated with preoperative characteristics and postoperative outcome [7, 8, 19, 24]. However, studies reporting recovery trajectories after revision TKA are lacking. In particular, the use of an anchor question - a single question where patients rate their change in health condition - is limited, while this measure is simple and often used in clinical practice to quantify a patient’s perception of their improvement or deterioration over time. A better understanding of recovery trajectories of patients undergoing revision TKA may lead to new insights and initiatives to improve preoperative consultation and postoperative rehabilitation of these patients. Consequently, this study aimed to describe various recovery trajectories after revision TKA. It has been suggested that PROMs are associated with range of motion in primary knee arthroplasty patients [23]. However, this is unclear in revision knee arthroplasty patients. Consequently, correlations between PROMs and range of motion were explored.

## Methods

A retrospective observational study was performed using data of a prospective revision cohort. Ethical approval was obtained from the local ethical committee (N15.103). This cohort comprises of data from a high volume center for knee revision surgery in the Netherlands. Demographic variables, surgical variables, prosthesis characteristics, range of motion, and PROMs were collected in this cohort that started in 2015. Informed consent was obtained by each patient in the study.

### Patient reported outcome measures

Health related quality of life, functional outcomes, pain and satisfaction were assessed using PROMs. These PROMs were assessed preoperatively, at three and 12 months postoperatively.

The descriptive system of the EuroQol-5 Dimensions (EQ-5D-3L) asks patients to value their general health status in five dimensions (i.e. mobility, self-care, usual activities, pain/discomfort, and anxiety/depression). The index score ranges from 0 (representing death) to 1 (representing full health), with negative values representing states worse than death [17]. Function and pain was measured using the Oxford Knee Score (OKS) with a range between 0 and 48 [6, 13], with higher scores on the OKS indicating less symptoms.

The Numeric Rating Scale (NRS) ranging from 0 to 10 was used to measure pain at rest and pain during activity, with higher scores indicating more severe pain [1, 18]. Satisfaction with the treatment was measured using the NRS ranging from 0 to 10, with higher scores indicating more satisfaction.

An anchor question, a subjective measure reflecting the patient’s point of view [4], was assessed three and 12 months after revision procedure. A change in physical functioning was assessed using a 7-point Likert scale. The response categories were: “very much worse”, “much worse”, “a little worse”, “about the same”, “a little better”, “much better”, and “very much better”.

### Patients and recovery trajectories

Patients undergoing a revision arthroplasty of the primary knee prosthesis between 2015 and August 2019 were included in this study. Revision procedures were performed by four senior orthopedic surgeons specialized in revision knee procedures. The range of revision systems form Smith & Nephew, such as the LEGION™ Revision Knee System (Smith & Nephew, London, United Kingdom) were used for revision knee arthroplasty. In two cases a rotating hinged Waldemar (Waldemar Link GmbH & Co. KG, Hamburg) was used. If possible, partial revision procedures were conducted (*n* = 40/88). Cemented fixation was used in 66% of the cases. Four groups were identified based on the anchor question regarding a change in physical functioning as compared to preoperatively (Table 1). Patients were excluded when this anchor question was not completed at both time points after revision procedure.

**Table 1:** Description of recovery trajectory groups

Recovery trajectory groups	Changes in physical functioning at three and twelve months after revision procedure as compared to preoperatively
No improvement group	Rated < 5 points on the anchor question at 3 and 12 months
Short improvement group	Rated ≥ 5 points on anchor question at 3 months and < 5 points at 12 months
Late improvement group	Rated < 5 points on the anchor question at 3 and ≥ 5 points at 12 months
Prolonged improvement group	Rated ≥ 5 points on anchor question at 3 and 12 months

*Note:* ≥ 5 indicates improvement

Eighty-eight participants were analyzed. Participant characteristics are described in Table 2. Sixty-nine percent of all participants (61/88) reported an improvement in physical functioning with the anchor question at 3 months. Sixty percent of all participants (53/88) reported an improvement in physical functioning with the anchor question at 12 months. The no improvement group comprised of 15 participants, short improvement group of 20 participants, late improvement group of 12 participants, and prolonged improvement group of 41 participants. Age was statistically different between the groups (*p* = 0.019). The short improvement group was significantly older as compared to the no improvement group (*p* < 0.001). In general, the most frequent cause of failure was malposition (33.0%), followed by aseptic loosening (21.6%), infection (14.7%), instability (11.4%), other (10.2%, e.g. pain, wear), and arthrofibrosis (9.1%). Differences in reason for revision were found between the groups (*p* = 0.040). Most frequent indication for revision was infection and instability for the no improvement group; arthrofibrosis for the short improvement group; aseptic loosening for the late improvement group; and malposition for the prolonged improvement group.

**Table 2:** Participant characteristics

	No improvement *n* = 15	Short improvement *n* = 20	Late improvement *n* = 12	Prolonged improvement *n* = 41
Age, years^*^	64 (7)	72 (4)	67 (10)	67 (9)
BMI, kg/m^2^	30 (6)	30 (5)	23 (4)	31 (7)
Male, n (%)	8 (53)	11 (55)	5 (42)	10 (24)
Smoking yes, n (%)	1 (6.7)	3 (15.0)	2 (16.7)	2 (4.9)
ASA I/II/III-IV, n	3/7/5	0/16/4	1/7/4	2/22/17
Charnley A/B1/B2/C/n.a., n	9/1/3/1/1	7/1/3/6/3	4/4/2/2/0	11/10/10/7/3
Time between index and revision procedure, months	24 (5–75)	35.5 (11–177)	53.5 (12–233)	45 (5–296)
Reason for revision, n (%)^*^				
Infection	4 (27)	2 (10)	1 (8)	6 (15)
Arthrofibrosis	2 (13)	4 (20)	1 (8)	1 (2)
Malposition	3 (20)	7 (35)	2 (17)	17 (42)
Aseptic loosening	0 (0)	5 (25)	5 (42)	9 (22)
Instability	4 (27)	1 (5)	0 (0)	5 (12)
Other	2 (13)	1 (5)	3 (25)	3 (7)

*Note:* presented as mean (standard deviation), number (percentage) or median (min-max). *BMI* body mass index, *ASA* American Society of Anesthesiologists physical status classification; ^*^, indicates significant difference between groups (*p* < 0.05)

### Statistics

The dataset contained 88 cases and 135 variables with 62 (71%) complete case observations. All variables were at least 94.3% complete. Data were assumed missing at random and five datasets were imputed. Missing values were imputed using fully conditional specification (IBM SPSS Statistics version 25). Predictive mean matching was used for the imputation of continuous variables. For categorical variables, imputation was done by logistic regression (binary outcome) or ordinal logistic regression (ordinal categorical with more than two levels). Distributions of imputed and observed values were compared and convergence across iterations was confirmed. All variables with missing values were included in the imputation models.

Repeated measures ANOVA was conducted for each PROM as dependent variable, and with recovery trajectories (group) and PROMs assessments (time) as independent variables. A Greenhouse-Geisser correction was used if sphericity was not met, and effect sizes (partial eta-squared) were calculated. The effect sizes were interpreted according to Cohen’s recommendation for 0.02 for a small effect, 0.13 for a medium effect, and 0.26 for a large effect [3]. Post-hoc analyses were conducted to assess differences between groups at each time point using Kruskal-Wallis Tests and Mann Whitney U tests as follow-up analysis. To assess improvement over time within each group, repeated measures ANOVA was conducted with post-hoc analyses to evaluate differences between two time points within each group. A Bonferroni correction was applied to all post-hoc analyses. Kruskall-Wallis Tests were used to assess differences in satisfaction with treatment and range of motion between groups. Change in range of motion at baseline compared to 12 months postoperative was assessed using Wilcoxon signed-rank *test*. Spearman correlation coefficients were calculated between range of motion and each PROM. The statistical significance level was set on 0.05.

## Results

### Quality of life

**Fig. 1:**
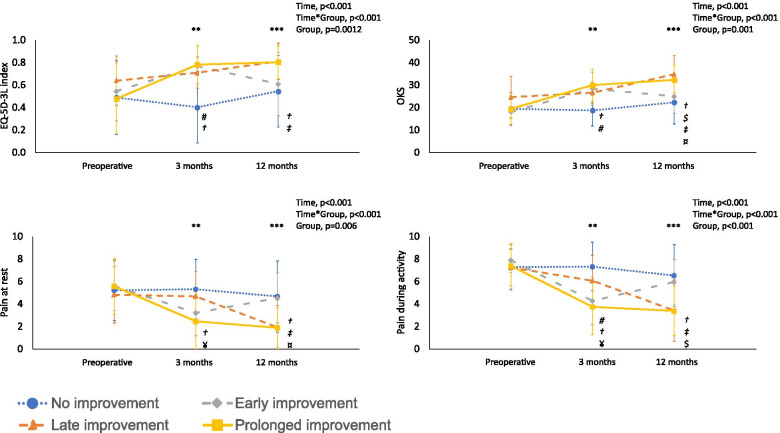
Patient Reported Outcome Measures of the recovery trajectory groups over time. A: EuroQol-5 Dimensions (EQ-5D-3L); B: Oxford Knee Score (OKS); C: pain at rest; D: pain during activity. Note: ^**^, significant differences between groups at 3 months postoperative; ^***^, significant differences between groups at 12 months postoperative; #, significant difference between No improvement group and Short improvement group; †, significant difference between No improvement group and Prolonged improvement group; ‡, significant difference between Short improvement group and Prolonged improvement group; ¤, significant difference between Short improvement group and Late improvement group; ¥, significant difference between Late improvement group and Prolonged improvement group; $, significant difference between No improvement group and Late improvement group

A significant interaction between group and time for EQ-5D-3L was found (*p* < 0.001, partial eta-squared = 0.188; Fig. 1A).

The groups did not differ in EQ-5D-3L before the revision procedure (*p* = 0.230). Differences between groups at three and 12 months after revision procedure were found (*p* < 0.001 and *p* = 0.001, respectively). EQ-5D-3L at 3 months after revision procedure was significantly higher in the short and prolonged improvement group as compared to the no improvement group (Fig. 1A; *p* = 0.008 and *p* < 0.001, respectively). The prolonged improvement group had significantly higher EQ-5D-3L at 12 months after revision procedure as compared to the no and short improvement group (Fig. 1A; *p* = 0.016 and *p* = 0.009, respectively).

EQ-5D-3L differed over time in the short, late and prolonged improvement group (all, *p* < 0.05). The short and prolonged improvement group reported a significantly higher EQ-5D-3L score at 3 months after revision procedure compared to preoperatively (Supplemental material 1, *p* = 0.014 and *p* < 0.001, respectively. The late and prolonged improvement group reported a significantly higher EQ-5D-3L score at 12 months after revision procedure as compared to preoperatively (Supplemental material 1; *p* = 0.025 and *p* < 0.001, respectively).

### Function and pain

A significant interaction between group and time for OKS was found (*p* < 0.001, partial eta-squared = 0.225; Fig. 1B).

The groups did not differ in OKS score before revision procedure (*p* = 0.270). Differences between groups at three and 12 months after revision procedure were found (both *p* < 0.001). OKS score at 3 months after revision procedure was higher in prolonged improvement group as compared to no improvement group (p < 0.001). Short improvement group reported a higher OKS score at 3 months as compared to no improvement group (*p* = 0.006). At 12 months after revision procedure, OKS score was higher in prolonged improvement group compared to no and short improvement group (*p* = 0.003 and *p* = 0.009, respectively). OKS score at 12 months after revision procedure was higher in late improvement group compared to no and short improvement group (*p* = 0.004 and *p* = 0.011, respectively).

OKS differed over time in the short, late and prolonged improvement group (all, *p* < 0.001). The short and prolonged improvement group reported a significantly higher OKS score at 3 months after revision procedure as compared to preoperatively (Supplemental material 1; *p* = 0.001; *p* < 0.001, respectively). The short, late, and prolonged improvement group reported a significantly higher OKS score at 12 months after revision procedure as compared to preoperatively (Supplemental material 1; *p* = 0.011; *p* = 0.001; and *p* < 0.001, respectively).

### Pain at rest

A significant interaction between group and time for pain at rest was found (p < 0.001, partial eta-squared = 0.200; Fig. 1C).

The groups did not differ in pain at rest before revision procedure (*p* = 0.790). Differences between groups at three and 12 months after revision procedure were found (both *p* < 0.001). Pain scores at 3 months after revision procedure, was lower in the prolonged group as compared to no and late improvement group (*p* = 0.001 and *p* = 0.025, respectively).

Prolonged improvement group reported lower pain scores at 12 months after revision procedure as compared to no and short improvement (*p* = 0.009 and *p* = 0.001, respectively). Late improvement group reported lower pain scores at 12 months after revision procedure as compared short improvement (*p* = 0.024).

Pain scores at rest differed over time in the short, late and prolonged improvement group (all, *p* < 0.001). The short and prolonged improvement group reported a significantly lower pain scores at rest at 3 months after revision procedure as compared to preoperatively (Supplemental material 1; *p* = 0.003 and *p* < 0.001, respectively). The late and prolonged improvement group reported a significantly lower pain at rest score at 12 months after revision procedure as compared to preoperatively (Supplemental material 1; *p* = 0.010 and *p* < 0.001, respectively).

### Pain during activity

A significant interaction between group and time for pain during activity was found (*p* < 0.001, partial eta-squared = 0.235; Fig. 1D).

Pain during activity did not differ between the groups before revision procedure (*p* = 0.742). Differences between groups at three and 12 months after revision procedure were found (both *p* < 0.001). Prolonged improvement group reported lower pain scores at 3 months after revision procedure as compared to no and late improvement group (*p* < 0.001 and *p* = 0.039, respectively). Short improvement group had lower pain scores during activity at 3 months after revision procedure as compared to no improvement group (*p* = 0.004).

Prolonged improvement group reported lower pain scores 12 months after revision procedure as compared to no and short improvement group (*p* = 0.001 and *p* = 0.002, respectively). Late improvement group had lower pain scores at 12 months after revision procedure as compared to no improvement group (*p* = 0.025).

Pain scores during activity differed over time in the short, late and prolonged improvement group (all, *p* < 0.001). The short and prolonged improvement group reported a significantly lower pain scores during activity at 3 months after revision procedure as compared to preoperatively (Supplemental material 1; both *p* < 0.001). The short, late, and prolonged improvement group reported significantly lower pain during activity at 12 months after revision procedure as compared to preoperatively (Supplemental material 1; *p* = 0.006; *p* = 0.001; and *p* < 0.001, respectively).

### Satisfaction & range of motion

Seventy-seven percent of all participants were satisfied with the treatment (≥6 points) at 3 months after revision procedure and 63% were satisfied at 12 months after revision procedure. Satisfaction with the outcome of the revision surgery differed between the groups at both time points (both *p* < 0.001; Table 3). The prolonged improvement group was more satisfied with the treatment at 3 months after revision procedure as compared to the late and no improvement group (*p* = 0.010 and *p* < 0.001, respectively). The prolonged improvement group was more satisfied with the treatment at 12 months after revision procedure as compared to no and late improvement group (both *p* < 0.001). The late improvement group was more satisfied with the treatment at 12 months after revision procedure as compared to no and short improvement group (*p* < 0.001 and *p* = 0.001, respectively). The range of motion did not differ between the groups at all three time points (Table 4). The range of motion improved between baseline and 12 months postoperative within the prolonged improvement group (*p* < 0.001). Range of motion at 12 months positive correlated with OKS scores at baseline in revision knee arthroplasty patients (*ρ* = 0.283, *p* = 0.011).

**Table 3:** Satisfaction with the outcome of revision knee arthroplasty

	No improvement *n* = 15	Short improvement *n* = 20	Late improvement *n* = 12	Prolonged improvement *n* = 41
Satisfaction with treatment at 3 months postoperative^*^	5 (0–10)	7 (0–10)	6.5 (4–8)	8 (5–10)^a^
Satisfaction with treatment at 12 months postoperative^*^	3 (0–10)	5 (0–8)^a^	8 (6–10)	8 (2–10)^a^

*Note:* presented as median (min-max). ^*^, indicates significant difference between groups (*p* < 0.05); ^a^, 1 missing value

**Table 4:** Range of motion values in recovery trajectory groups

	No improvement *n* = 15	Short improvement *n* = 20	Late improvement *n* = 12	Prolonged improvement *n* = 41
RoM preoperative	105 (45–140)	120 (50–140)	120 (75–140)	115 (65–140)
RoM 3 months postoperative	115 (50–140)^a^	120 (85–130)^b^	115 (95–130)	120 (100–130)^a^
RoM 12 months postoperative	110 (50–130)^c^	120 (40–135)^b^	120 (105–135)^a^	120 (100–135)^a*^

*Note:* presented as median (min-max). *RoM* range of motion; ^a^, 1 missing value; ^b^, 2 missing values; ^c^, 3 missing values; ^*^, indicates significant improvement preoperative versus twelve months postoperative (*p* < 0.05)

## Discussion

This study describes clinical outcomes of four recovery trajectories of knee revision arthroplasty patients. Overall 60% of revision TKA patients reported improved physical functioning at 12 months after revision procedure and 63% percent was satisfied with the overall result at 12 months after revision procedure. Recovery trajectory groups demonstrated different trajectories in terms of quality of life, function, and pain within 12 months after knee revision procedure. PROM trajectories of the no, late and prolonged improvement group were in line with answers on the anchor questions at three and 12 months after revision procedure. The short improvement group, however, did not report an improvement in physical functioning at 12 months while OKS and pain scores improved statistically. Level of improvement in OKS and pain scores was larger than the available minimal clinical important difference for these PROMs suggesting that improvement in experienced pain and function was meaningful for the patients [2, 9]. This finding might indicate a mismatch between the anchor question and validated PROMs for a sub selection of patients. In addition, the range of motion seems to be none to weakly associated with PROMs. In general, this study provides insight into different recovery trajectories after revision knee arthroplasty, which could be used for counseling patients about the expectations and management of revision knee arthroplasty outcomes.

This study showed that PROMs differed over time between the recovery trajectory groups. Though quality of life, function or pain scores did not significantly differ between the groups before knee revision procedure, differences between the groups were found at three and 12 months postoperatively. These findings indicates that patients react differently during their recovery period within the first year after revision knee arthroplasty. Possible explanations for different recovery trajectories may lie in patient characteristics, such as age, gender, preoperative function scores and indication for revision [15, 33, 35]. In this study, age differed significantly between the groups, in particular between the no improvement group and short improvement group. This study observed differences in indication for revision between the groups, which is in agreement with studies reporting that indication for revision is an important factor in clinical outcomes [26, 33, 34]. This highlights the importance of future studies on identifying patient groups and their explanatory or predictable factors for recovery to further improve and personalize patient care.

Previous studies on clinical outcomes after revision knee arthroplasty have found.

improvements in function and pain scores [15, 26, 30, 33, 35]. In addition, postoperative quality of life demonstrated good scores after knee revision arthroplasty [28]. Similar improvements in postoperative clinical outcomes were found in the current study. Improvement in quality of life at 12 months after revision procedure reached the minimal clinical important ranging from 0.03 to 0.52 [4] for the all groups except the no improvement group. In addition, improvements in function at 12 months after revision procedure met the minimal clinical important difference for the OKS of five points [2] in the short, late and prolonged improvement group. Improvement in pain at rest and during activity at 12 months after revision procedure met the minimal clinical important difference of two points [9] in the late and prolonged improvement group. These results support the idea that revision TKA is beneficial for patients. It should, however, be noted that not all patient experience beneficial outcome of revision TKA. It is therefore important to thoroughly inform the patient on the expected course of recovery and outcome after revision TKA.

A weak correlation was found between the range of motion at 12 months postoperative and the Oxford Knee Score at baseline. This is in line with Padua et al. [23], reporting weak to moderate correlations between range of motion and several domains of the Short Form 36 and the Oxford Knee Score in primary knee arthroplasty patients. However, our finding should be interpreted with caution as the PROMs were assessed in a different patient population and only one correlation was found statistically significant.

A main consideration for a revision procedure is based on indications, which differs between patients. Surgical interference is appropriate for most indications, in which patients can decide on whether or not to undergo a revision procedure. However, this is different in case of a periprosthetic joint infection, there is less of choice due to the negative consequences of an ongoing periprosthetic joint infection. Clinical outcomes of revision TKA may not be as successful in patients with infection as those in patients without infection [27]. In the present study, periprosthetic joint infection as indication for revision was divided over all groups (no improvement *n* = 4; short improvement *n* = 2; late improvement *n* = 1; prolonged improvement *n* = 6). When periprosthetic joint infection patients were excluded from the analyses, similar results considering clinical outcome over time and differences between groups were found. In addition, arthrofibrosis may result in disability among patients due to knee pain and restricted range of motion than can hinder rehabilitation and activities in daily life [10]. As arthrofibrosis seems to be a more frequent indication for revision in the no and short improvement groups as compared to the late and prolonged improvement groups, this indication may be associated with less improvements in clinical outcomes.

There are several limitations that need to be addressed. The limitations mainly concern the design of this study, in which retrospective analyses with its corresponding bias and missing data were conducted. Missing data were, however, addressed using multiple imputation. Another limitation is, that patients who did not complete both anchor questions regarding physical functioning were excluded from the analyses. These patients might not have completed all anchor questions due to, for example another revision procedure during their first year or were lost to follow up. As a result, 88 (73.3%) of all revision TKA (*n* = 120) of the study period were included. However, there were no differences in patient characteristic between included and excluded patients suggesting we were able to present data of a representative cohort. Although the findings in this single center study might be less generalizable to other centers. This study however was conducted at a high volume center for revision surgery, which is comparable in type of revision procedures to other high volume centers in the Netherlands. Due to the complexity of revision procedures and their impact on quality of care, these procedures should be centralized in specialized centers.

Clinical outcome after revision knee arthroplasty seems to differ between four recovery trajectory groups. Sixty percent of the revision population reported improvement in physical functioning. In the majority of the patients, this was linked to improved quality of life, function and pain scores at 12 months after revision procedure. Different trajectories, however, seem to exist and not all patients may experience beneficial outcome of revision TKA. These findings are of importance to provide appropriate honest information to patients about the possible recovery trajectory after revision knee arthroplasty.

## Supplementary Information

**Additional file 1.**

## Data Availability

The dataset used and/or analysed during the current study are available from the corresponding author on reasonable request.

## Abbreviations

PROMs: Patient reported outcome measures
OKS: Oxford Knee Score
EQ-5D-3L: EuroQol EQ-5D-3L
NRS: Numeric Rating Scale
TKA: Total knee arthroplasty
BMI: Body mass index
ASA: American Society of Anesthesiologists physical status classification
