# Supplementary material for: Recovery patterns in patients undergoing revision surgery of the primary knee prosthesis
Source: J Exp Orthop. 2021 Dec 16;8:117. doi: 10.1186/s40634-021-00436-w (PMC8674388; doi:10.1186/s40634-021-00436-w)
Supplement: Supplementary file 1 — Additional file 1. [file 40634_2021_436_MOESM1_ESM.docx]

**Supplemental material 1**

**Table 1** Patient Reported Outcome Measures of recovery trajectory groups

|  | | **EQ-5D-3L** | | | | **OKS** | | | | **NRS pain at rest** | | | | **NRS pain during activity** | | | |  |  |
| --- | --- | --- | --- | --- | --- | --- | --- | --- | --- | --- | --- | --- | --- | --- | --- | --- | --- | --- | --- |
|  | | T0 | T3 | T12 | p | T0 | T3 | T12 | p | T0 | T3 | T12 | p | T0 | T3 | T12 | p |  | |
| No improvement | Mean (SD) | 0.49 (0.33) | 0.40 (0.32) | 0.55 (0.32) |  | 19.53 (7.23) | 18.82 (7.11) | 22.27 (9.66) |  | 5.20 (2.68) | 5.33 (2.64) | 4.67 (3.16) |  | 7.27 (2.02) | 7.33 (2.16) | 6.53 (2.75) |  |  |  |
| Short improvement | Mean (SD) | 0.55 (0.26) | 0.77 (0.08) | 0.61 (0.28) | ^†/^* | 18.08 (6.20) | 28.45 (7.32) | 24.90 (7.40) | ^†/*/**^ | 5.60 (2.22) | 3.20 (2.02) | 4.55 (2.24) | ^†/*^ | 7.88 (1.05) | 4.30 (2.13) | 5.95 (2.01) | ^†/*/**^ |  |  |
| Late improvement | Mean (SD) | 0.64 (0.22) | 0.71 (0.14) | 0.81 (0.16) | ^†/**^ | 24.67 (9.31) | 26.67 (4.77) | 34.75 (8.38) | ^†/**/***^ | 4.83 (2.52) | 4.67 (2.23) | 1.92 (1.93) | ^†/*/***^ | 7.25 (1.60) | 6.08 (2.23) | 3.42 (2.75) | ^†/*/***^ |  |  |
| Prolonged improvement | Mean (SD) | 0.48 (0.31) | 0.78 (0.17) | 0.80 (0.15) | ^†/*/**^ | 19.37 (6.76) | 29.92 (7.08) | 32.25 (6.72) | ^†/*/**^ | 5.58 (2.47) | 2.46 (2.44) | 1.90 (1.76) | ^†/*/**^ | 7.38 (1.98) | 3.76 (2.49) | 3.39 (2.19) | ^†/*/**^ |  |  |

*Note:* EQ-5D-3L, EuroQol; T0, indicates preoperative; T3, indicates three months postoperative; T12, indicates twelve months postoperative; ^†^, indicates significant difference over time; ^*^, indicates significant differences between before and three months after revision procedure (p <0.05). ^**^, indicates significant differences between before and twelve months after revision procedure (p <0.05); ^***^, indicates significant differences between three months and twelve months after revision procedure (p <0.05).
